# Nitrous oxide and nitric oxide emissions from lowland rice cultivation with urea deep placement and alternate wetting and drying irrigation

**DOI:** 10.1038/s41598-018-35939-7

**Published:** 2018-12-04

**Authors:** S. M. Mofijul Islam, Yam Kanta Gaihre, Jatish Chandra Biswas, Upendra Singh, Md. Nayeem Ahmed, Joaquin Sanabria, M. A. Saleque

**Affiliations:** 1 Bangladesh Rice Research Institute, Soil Science Division, Gazipur, Bangladesh; 2 International Fertilizer Development Center, Dhaka, Bangladesh; 3 International Fertilizer Development Center, Muscle Shoals, Alabama USA; 4 International Rice Research Institute, Dhaka, Bangladesh

## Abstract

Urea deep placement (UDP) and the alternate wetting and drying (AWD) irrigation method are two promising rice production technologies. However, studies on the impact of UDP under AWD irrigation on nitrous oxide (N_2_O) and nitric oxide (NO) emissions are limited. In this study, the effects of UDP with AWD irrigation on these emissions, nitrogen use efficiency (NUE), and rice yields are investigated, compared to conventional broadcast application. N_2_O and NO emissions from three fertilizer treatments – no nitrogen, UDP, and broadcast application of prilled urea (PU) – were measured. Measurements were taken using an automated gas sampling and analysis system continuously for two consecutive Boro (dry) rice seasons. N_2_O emission peaks were observed after broadcast application of PU but not after UDP. In contrast, large spikes in N_2_O emission were observed after UDP, compared to broadcast application, during dry periods. Despite differences in emission peaks, seasonal cumulative N_2_O emissions from UDP and broadcast treatments were similar. However, NO emissions were minimal and unaffected by UDP or AWD. UDP increased rice yields by 28% and N recovery efficiency by 167%, compared to broadcast urea. This study demonstrates that UDP with AWD irrigation can increase yields and NUE without increasing N_2_O and NO emissions.

## Introduction

Agriculture is a significant anthropogenic source of nitrous oxide (N_2_O) and nitric oxide (NO), contributing up to 60% of total anthropogenic N_2_O emissions^1,2^. N_2_O emission is a major concern due to its increasing concentration in the atmosphere. N_2_O concentration has increased from 270 ppb during pre-industrial times to 328.9 ppb in 2016^3^. The average annual carbon dioxide (CO_2_)-equivalent emission was 52 gigatons (Gt) in 2011. This is expected to almost double by 2050 if mitigation actions are not accelerated^2,4^. N_2_O is a strong greenhouse gas (GHG) that is 265 times more potent than CO_2_ over a 100-year time horizon^4^. In addition to trapping heat in the atmosphere, N_2_O contributes to the destruction of the stratospheric ozone layer^5^. N_2_O alone contributed 6% to total anthropogenic GHG emissions in 2010^4^. Since both N_2_O and NO gases are produced in the soil through the biochemical process of nitrification and denitrification^6,7^, their emissions are highly variable with soil properties, climate, crops, irrigation regimes, and fertilizer sources and methods of application^8–10^. NO contributes to acidification and eutrophication of ecosystems and plays an important role in the formation of ozone in the lower atmosphere^7,11^.

In agriculture, N_2_O production is associated mainly with nitrogen (N) fertilizer application. Microbial nitrification and denitrification are major processes affecting N_2_O and NO emissions and N availability to plants. However, there are other processes producing N_2_O, such as chemical decomposition of hydroxylamine, chemodenitrification, and abiotic decomposition of ammonium nitrate^12^. Nitrification is an aerobic oxidation of ammonium (NH_4_^+^) to nitrate (NO_3_^−^) in a two-step process in which NH_4_^+^ is oxidized to nitrite (NO_2_^−^) by primary nitrifiers (*Nitrosomonas sp*.) and NO_2_^−^ is oxidized to NO_3_^−^ by secondary nitrifiers (*Nitrobacter sp*.). Alternatively, denitrification is the stepwise reduction of NO_3_^−^ to N_2_, mediated by facultative anaerobic bacteria in an oxygen-limiting environment^12^. Details of the nitrification and denitrification processes are given in Butterbach-Bahl *et al*.^12^. The magnitude of N_2_O emission from soil depends on the amount of N fertilizer applied, the availability of NH_4_^+^ and NO_3_^−^ in soils, soil moisture, and temperature. In addition to the quantity of fertilizer applied, the N application method affects emissions. Nitrogen use efficiency (NUE) of broadcast-applied prilled urea (PU) in lowland rice fields is only 30–40%^13,14^ due to losses through ammonia (NH_3_) volatilization, surface runoff, nitrification, denitrification, and leaching^13,15^. The amount of N loss due to volatilization increases as long as the floodwater pH and temperature are favorable^16^. Broadcast application of PU produces higher floodwater NH_4_^+^-N compared to deep placement of N^17–21^. This increases the loss of N as NH_3_ volatilization, which increases with increasing N rates. Therefore, the excessive use of N fertilizer via broadcast application has negative environmental consequences as a result of N_2_O and NO emissions, nitrate pollution of groundwater, and eutrophication^13^.

Research in the past decades has focused on N management strategies, including the right fertilizer source, right rate, proper application timing, and methods of application, to increase crop productivity and NUE while reducing the negative environmental consequences. Increasing NUE has been achieved by using nitrification inhibitors, urease inhibitors, slow-release fertilizers, balanced crop nutrition^22^, and improved placement methods. Efficient fertilizer products and placement methods were found effective in mitigating N_2_O and NO emissions^23,24^. Urea deep placement (UDP) is among the best N management strategies and a currently applicable technique, as it increases rice yields up to 20% and saves urea use up to 30%^13,18,20,25^, compared to surface application of PU.

In addition to N management, water management plays an important role in N_2_O and NO emissions from rice fields^8,26^. For example, continuous flooded rice cultivation emits less N_2_O^23,25,26^, because in water-saturated and anaerobic conditions, much of the N_2_O is reduced to N_2_ by denitrifiers before escaping from the soil^7,12^. In saturated soils, deep-placed N remains in the root zone as NH_4_^+^-N, which is less subject to nitrification. This can ensure a continuous supply of N to plants throughout the growing season, depending on soil type. In contrast, alternate wetting and drying (AWD) irrigation triggers considerable N_2_O and NO emissions^27–29^. The intermittent soil drying and wetting often produces cracks, particularly in clayey soil, in addition to changes in soil physicochemical properties^30^. These cracks increase the oxygen content in the deeper soil layer, which in turn increases the rate of nitrification and, thus, N_2_O emissions^30,31^. Unlike continuously flooded conditions, alternating the soil between dry and wet states makes soil N unstable, as it favors nitrification and subsequent denitrification, leading to more N_2_O and NO emissions^12,32,33^. Therefore, the addition of N fertilizer, either as broadcast or deep placement, under AWD irrigation may increase the conversion rate of soil NH_4_^+^ to NO_3_^−^ as well as the rate of subsequent denitrification of the NO_3_^−^, leading to more N_2_O emissions^12,27,34^.

In Bangladesh, the groundwater table is depleting due to continuous pumping for Boro rice (dry season, between December/January and April/May) cultivation. This leads to increased irrigation water pumping costs for farmers^35,36^. Adoption of the AWD irrigation practice has been shown to reduce water use by up to 38% without significant yield penalty^35^. Similarly, UDP has been gaining popularity for rice cultivation in some Asian countries due to saving fertilizer and increasing NUE and grain yields^17,36^. In our previous study^24,37^, UDP significantly reduced N_2_O emissions compared to broadcast-applied PU, particularly when rice was cultivated with continuous flooding irrigation. Most previous studies with UDP have been conducted under conventional irrigation practices, i.e., continuous standing water (CSW) conditions. A limited number of studies have been performed with UDP under AWD conditions; these studies reported effects of UDP on grain yields and NUE^18–20^, indicating that UDP increases NUE and grain yields, compared to PU, in both AWD and CSW conditions. However, no studies have been conducted with UDP under AWD irrigation to determine the impact on N_2_O and NO emissions. UDP under AWD irrigation is hypothesized to be equally effective in terms of yield, NUE, and mitigation of emissions of N_2_O and NO as under CSW conditions. Therefore, the present field study was conducted to investigate the effects of UDP on N_2_O and NO emissions, grain yields, and NUE under AWD conditions during two consecutive Boro seasons.

## Results

### Floodwater ammonium nitrogen (NH_4_^+^-N)

**Figure 1:**
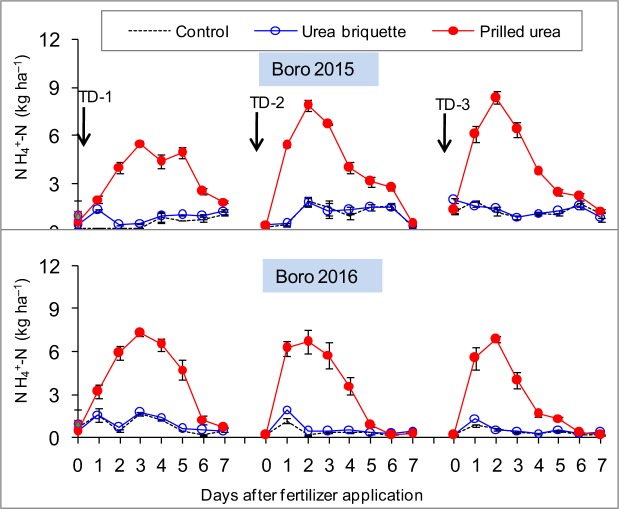
Dynamics of floodwater NH_4_^+^-N in control, urea briquette, and PU treatments under alternate wetting and drying conditions in dry (Boro) season. TD-1, TD-2, and TD-3 represent first, second, and third topdressing of PU, respectively. Vertical bars indicate standard error of mean (n = 3).

The dynamics of floodwater NH_4_^+^-N were measured during seven consecutive days after N fertilizer application in two rice-growing seasons (Fig. 1). Deep placement of urea briquettes significantly reduced floodwater NH_4_^+^-N, compared to broadcast PU. Peaks of floodwater NH_4_^+^-N with the PU treatment were observed two to three days after fertilizer application, followed by a sharp decline, while treatment with urea briquettes showed floodwater NH_4_^+^-N similar to the control treatment throughout the measurement period. The magnitudes of floodwater NH_4_^+^-N were consistent in both years.

### Seasonal dynamics of N_2_O emissions

**Figure 2:**
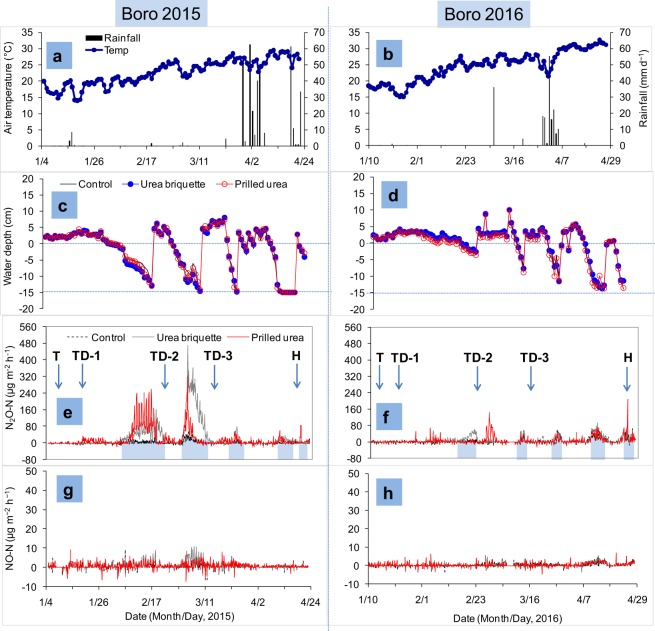
Nitrous and nitric oxides emission rates, floodwater depth, air temperature, and rainfall under AWD conditions during Boro 2015 and Boro 2016 seasons at BRRI, Gazipur. T, TD-1, TD-2, TD-3, and H correspond to transplanting, first topdressing, second topdressing, third topdressing, and harvesting, respectively. The shaded area in the x-axis indicates the drying period.

The dynamics of N_2_O emissions from the control, UDP, and broadcast PU treatments are presented in Fig. 2e,f. N_2_O emissions peaks were observed after topdressing of PU, during the drying period, or after re-flooding of dry soil. Except during the peak emission events, N_2_O emissions were within a range of ±30 µg N m^−2^ h^−1^, irrespective of treatment and season.

In the Boro 2015 season, N_2_O emission peaks were observed after two to four days for each topdressing of PU and continued for up to eight days (Fig. 2e). In contrast, emission peaks were not observed in the UDP treatment except during the dry period. Though large spikes of N_2_O-N emission were observed during the dry period in both treatments, the magnitudes of the emission were more prominent (466 µg N m^−2^ h^−1^) and appeared for a longer time in the UDP treatment, compared to broadcast PU (324 µg N m^−2^ h^−1^). In Boro 2016, N_2_O emission peaks were observed with broadcast PU three days after the second topdressing and continued consistently for eight days. In contrast, the N_2_O emission peaks were very small to negligible after the first and third topdressing of PU (Fig. 2f). As in Boro 2015, peaks of N_2_O emission were observed with both treatments during each dry period, but they were not as prominent as in Boro 2015. In general, drying-induced emissions were more prominent and prolonged compared to fertilizer-induced emissions. Occasional negative fluxes were observed with all treatments, but these did not show any consistent pattern.

### Seasonal dynamics of NO emissions

NO emission rates from all treatments are displayed in Fig. 2g,h. Occasional emission peaks were observed after topdressing of PU and during the drying period. The impacts of fertilizer placement methods on NO emission rates were not consistent. However, during dry episodes, the magnitudes of emission were slightly higher with the UDP treatment than broadcast PU, particularly in 2015. NO emissions were relatively higher in Boro 2015, compared to Boro 2016. Sporadic negative NO emissions were observed with all treatments, particularly during midnight through early morning periods. Irrespective of treatment, NO fluxes varied from −7 µg N m^−2^ h^−1^ to 11 µg N m^−2^ h^−1^ in Boro 2015 and from −4 µg N m^−2^ h^−1^ to 7 µg N m^−2^ h^−1^ in Boro 2016.

### N_2_O emissions: Seasonal total and yield-scaled emissions

Fertilizer treatment had significant interaction effects with year on cumulative and yield-scaled emissions (Table 1). In 2015, cumulative and yield-scaled emissions ranged from 63 g N ha^−1^ to 732 g N ha^−1^ and from 40 g t^−1^ grain to 153 g t^−1^. In Boro 2016, they ranged from 116 g N ha^−1^ to 209 g N ha^−1^ and from 51 g N t^−1^ grain to 41 g N t^−1^ grain (Table 1). In 2015, the addition of N fertilizer, irrespective of method of application, increased cumulative emissions significantly (p < 0.05), compared to the control treatment. However, the difference between UDP and PU was not significant. Similar results were observed for yield-scaled emissions. In 2016, no significant differences were observed on cumulative and yield-scaled N_2_O emissions among the treatments. Fertilizer-induced emission factors (EFs) were 0.42% and 0.86% in 2015 and 0.05% and 0.12% in 2016 for PU and UDP treatments, respectively (Table 1). The effects of fertilizer placement method on EF were insignificant (p > 0.05). The EFs were significantly higher in 2015 compared to 2016.

**Table 1:** Seasonal N_2_O-N emissions, yield-scaled emissions, and emission factors with different N fertilizer treatments under AWD conditions, BRRI, Gazipur.

Treatment	N_2_O-N emission (g ha^−1^ season^−1^)		Yield-scaled N_2_O-N emission (g t^−1^ grain)		N_2_O-N emission factor (%)	
	2015	2016	2015	2016	2015	2016
Control	63.30bA	115.94 aA	40.17bA	51.34 aA	—	—
Urea briquette	731.55 aA	209.31aB	152.98 aA	39.69aB	0.857 aA	0.120aB
Prilled urea	500.96 aA	162.31aB	136.61 aA	40.72aB	0.421 aA	0.045 aA
**Mean**	**431.94A**	**162.52B**	**109.91A**	**43.92B**	**0.639A**	**0.082B**
Control	89.62b		45.75b		—	
Urea briquette	470.43a		96.33a		0.488a	
Prilled urea	331.64a		88.66a		0.233a	
**ANOVA (p values)**						
Year (Y)	0.0022		0.0014		0.0036	
Treatment (T)	0.0036		0.0326		0.0994	
Y × T	0.0145		0.0117		0.2248	

Note: In a column, means followed by the same lowercase letters and, in a row for each response variable, means followed by the same uppercase letters are not significantly different at a 5% level of probability by Tukey’s honest significant difference (HSD) test.

### NO emissions: Seasonal total and yield-scaled emissions

Cumulative and yield-scaled NO emissions were very low compared to N_2_O emissions. The effects of fertilizer treatments and their interaction with year were not significant (p > 0.05). Across the seasons, they ranged from 12.23 g N ha^−1^ to 14.73 g N ha^−1^ and from 3.78 g t^−1^ grain to 6.80 g t^−1^ grain (Table 2). Similarly, the fertilizer-induced EF was not affected by the method of N fertilizer application or its interaction with year (Table 2). Across the seasons, fertilizer-induced EFs were 0.009% and 0.003%, respectively, for UDP and PU treatments (Table 2). However, NO EF was significantly (p < 0.05) higher in 2015 than in 2016.

**Table 2:** Seasonal NO-N emissions, yield-scaled emissions, and emission factors (two-year average) with different N-fertilizer treatments under AWD conditions, BRRI, Gazipur.

Treatment	NO-N emission (g ha^−1^ season^−1^)	Yield-scaled NO-N emission (g t^−1^ grain)	NO-N emission factor (%)
Control	12.23a	6.80a	—
Urea briquette	18.08a	3.89a	0.009a
Prilled urea	14.72a	3.78a	0.003a

Note: In a column, means followed by the same letters are not significantly different at a 5% level of probability by Tukey’s honest significant difference (HSD) test.

### Grain yield and NUE

Across the seasons, UDP significantly increased grain yield by 28% compared to broadcast PU (Table 3). Similarly, both agronomic use efficiency (AE_N_) and recovery efficiency (RE_N_) of N were significantly affected by method of N fertilizer application (Table 3). UDP increased AE_N_ and RE_N_ by 109% and 167%, respectively, compared to broadcast PU. There were no significant interaction effects between treatment and year for studied variables (Table 3).

**Table 3:** Effects of N application method on grain yields, agronomic use efficiency, and recovery efficiency of N (two-year average) under AWD conditions, BRRI, Gazipur.

Treatment	Grain yield (t ha^−1^)	AE_N_ (kg grain N kg^−1^)	RE_N_ (kg N uptake N kg^−1^)
Control	1.91c	—	—
Urea briquette	4.95a	38.88a	0.56a
Prilled urea	3.86b	18.59b	0.21b
**ANOVA (P Values)**			
Year (Y)	0.0024	0.2292	0.4280
Treatment (T)	<0.0001	<0.0001	<0.0001
Y × T	0.5174	0.6267	0.5512

Note: In a column, means followed by the same letters are not significantly different at a 5% level of probability by Tukey’s honest significant difference (HSD) test.

## Discussion

N fertilizer application method and water regime significantly influence N_2_O emissions. In this study, N_2_O emission from the UDP treatment tended to be higher (by an average of 37%) than broadcast PU, but this difference was below statistical significance (p > 0.05) (Table 1). In contrast to these observations, Gaihre *et al*.^37^ reported that UDP reduced cumulative N_2_O emissions significantly, compared to broadcast PU; however, in that study, experiments were conducted under a CSW irrigation regime in which emissions from UDP were very low, relative to the present study in which experimental plots were maintained under AWD conditions. Higher emissions from the UDP treatment could be due to AWD irrigation. During the dry period, higher N_2_O emission peaks were observed with UDP compared to broadcast PU (Fig. 2e,f). In general, the UDP plot retained more NH_4_^+^-N in the soil (less in the floodwater) (Fig. 1). Drying may have increased microbial nitrification, while re-flooding increased denitrification, resulting in more N_2_O emissions^28,32–34^. Since UDP in flooded soils significantly reduces floodwater NH_4_^+^-N (Fig. 1), most of the N is retained as NH_4_^+^ in a reduced zone for an extended period of time, and a very small amount of N is lost through NH_3_ volatilization, nitrification, denitrification, and leaching^13,15,21^. As a result, UDP can supply available N to the plant throughout the rice-growing season^13,21^. With drying of the plot, NH_4_^+^ retained in the sub-surface layer could be oxidized to NO_3_^−^ (nitrification including chemical decomposition of hydroxylamine)^12^, resulting in greater N_2_O emissions.

In the case of PU, application was broadcast; this led to more floodwater NH_4_^+^-N (Fig. 1), which increased soil pH instantly. Subsequently, the NH_4_^+^-N was rapidly lost through NH_3_ volatilization, surface runoff, leaching, and nitrification-denitrification^13,21,38^. However, N_2_O emissions are not restricted to N fertilizer application sites (direct emissions); rather, N cascading from application sites (via volatilization, leaching, and erosion) to downwind and downstream ecosystems may result in natural ecosystem N enrichment, thereby creating new hot spots of N_2_O emissions, called indirect emissions. Therefore, comparatively lower N_2_O emission peaks were observed from broadcast PU during the dry period, due to less soil NH_4_^+^. Nevertheless, the difference in cumulative N_2_O emissions between UDP and PU was not significant (Table 1). Except after topdressing of PU and during the dry period, N_2_O emissions were very low to negligible when the field was continuously flooded. In general, N_2_O emission is negligible in continuously flooded paddy fields^27,28^, because N_2_O is further reduced to N_2_ during denitrification^6,12,27^. Occasional negative fluxes were observed in the present study, which is consistent with previous reports^24,37,39–41^. The negative fluxes, essentially the uptake of N_2_O by soil, tend to occur under conditions of low soil inorganic N and high soil water content^39^. Under such conditions, N_2_O could be further reduced to N_2_, particularly during denitrification.

**Figure 3:**
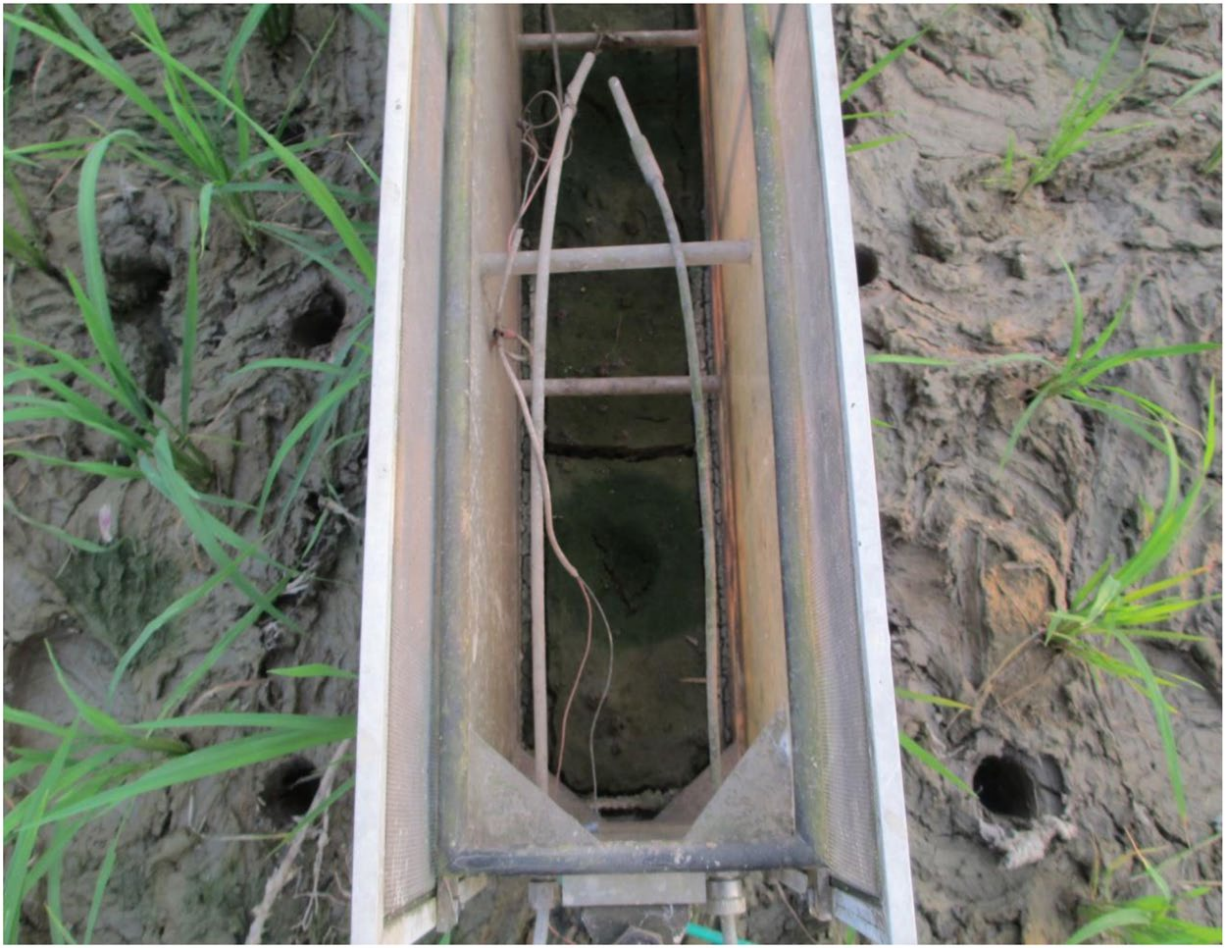
Soil cracks inside the gas chamber during the drying period.

Large seasonal variations in N_2_O emission were observed in this study (Table 1), which is consistent with previous reports^24,32,37,42^. Higher N_2_O emission peaks and cumulative emissions, particularly in fertilized plots, were observed in 2015 compared to 2016 (Fig. 2e,f); these peaks were associated with the drying intensity of the soil. In 2015, when floodwater depth dropped close to or below 15 cm from the soil surface, the upper layer of soil produced large cracks (Fig. 3). This increased soil aeration, favoring microbial nitrification, and resulted in higher N_2_O emissions^43^. In contrast, in 2016, the water level did not drop as rapidly, and dry episodes were much shorter. These results suggest that variations in the magnitude of N_2_O emissions depend on the intensity of soil drying and wetting, rather than on season or year.

The application of N fertilizer significantly increased yield-scaled N_2_O emissions, compared to the control treatment in 2015, but UDP and broadcast PU showed comparable yield-scaled N_2_O emissions in both seasons (Table 1). However, UDP reduced emission intensity (g N_2_O t^−1^ grain), compared to cumulative emissions, as UDP had significantly higher grain yields over broadcast PU (Table 3). Although there were no additional benefits by reducing significant amounts of cumulative N_2_O and yield-scaled N_2_O emissions from the UDP treatment over PU under AWD irrigation, UDP significantly increased rice grain yields and NUE. Previous studies reported that UDP with CSW irrigation significantly reduced cumulative N_2_O emissions and yield-scaled emissions, compared to broadcast PU^24,37^. Our results suggest that UDP under AWD irrigation is equally effective as under CSW, since it increased grain yields and NUE without increasing N_2_O and NO emissions.

Across treatments and seasons, the fertilizer-induced N_2_O EF was 0.36% (Table 1). Similar EFs of N_2_O during the rice-growing season have been reported in previous studies^44,45^. The mean fertilizer-induced EF of N_2_O for fertilized rice fields under midseason drainage was 0.37%, as reported by Akiyama *et al*.^32^, which is similar to findings in this study. The fertilizer-induced EF for UDP varied significantly with the season, which may be attributed to seasonal cumulative N_2_O emissions (Table 1). However, our EF values are less than the Intergovernmental Panel on Climate Change (IPCC) default EF (1.0%)^46^ for the entire rice-growing season.

Emission peaks of NO were observed after N fertilizer application, but they were very small and inconsistent, compared to N_2_O peaks, even in the dry period (Fig. 2g,h). Increases in NO emissions after N fertilizer application have been observed not only in rice^28^ but also in vegetables^47^. NO gas is produced mainly from oxidized soil through nitrification, although both nitrification and denitrification can produce NO^48,49^. The effect of N fertilizer on the magnitude of NO emission peaks is largely influenced by soil type, soil moisture, soil temperature, fertilizer type, and ambient NO concentration^50,51^, particularly under oxidized field conditions. Since dry-wet alternation favors both nitrification and denitrification, greater NO emission peaks were produced with the UDP treatment. The presence of more NH_4_^+^ with UDP in sub-surface soil, particularly during the dry period, may have provided more substrate for prolonged nitrification. Previous studies have also reported higher NO emission under AWD conditions^29,52^. In this study, although UDP showed large spikes of NO emission under wet-dry alternation compared to broadcast PU, cumulative emissions with UDP and broadcast PU were similar (p > 0.05). The lower NO emissions may be associated with its diffusion from production sites. NO is produced mainly in the fertilized layer and placement site but does not diffuse from the production sites because of its rapid uptake in the soil^53,54^. Nevertheless, these results confirm that NO emission from rice fields with soil, climatic, and N management conditions similar to the present study site is negligible, regardless of irrigation regime and fertilizer placement method^24,37^.

Significant variations in yield-scaled NO emissions between broadcast and deep-placed treatments were not observed; however, grain yields varied between application types (Tables 2 and 3). Similarly, fertilizer-induced EFs were not affected by N placement method; EF values were very low, compared to 0.7% of applied N reported by Bouwman *et al*.^55^ and 0.04% in rice fields in China^56^. The lower EF of NO in the present study is probably associated with higher background emissions due to the urban and industrial nature of the area surrounding the study site. Notably, our previous study^24,37^ in the same area under CSW conditions showed similar results.

Application of N fertilizer significantly increased grain yields in both seasons (Table 3). The use of UDP with 25% less urea significantly improved rice yields and resulted in a marked increase in NUE (AE_N_ and RE_N_), compared to broadcast PU. Ostensibly, UDP increased the contact of fertilizer molecules with soil particles and reduced NH_4_^+^-N in floodwater (Fig. 1), while also reducing NH_3_ volatilization. In general, UDP prolongs available N supply throughout the rice-growing season^17,57^, thereby promoting total N uptake and, ultimately, improving NUE and grain yield (Table 3). Results from this study are in close agreement with previous studies conducted under continuous flooding conditions^17,57^, which reported that UDP significantly increased rice yield by 15–20% and urea savings by 25–50%, compared to broadcast application of PU. Therefore, these results confirm that UDP is effective under both CSW and AWD irrigation regimes.

In addition to saving N fertilizer and increasing NUE and grain yields, UDP with AWD irrigation saves water by up to 38%^35^. Adoption of AWD irrigation, however, increases N loss as N_2_O emissions, compared to continuous flooding irrigation. In this study, the average (two-year) N losses as N_2_O from UDP and broadcast PU treatments were 0.49% and 0.23% of applied N, respectively, which are higher compared to losses that occurred with continuous flooding irrigation (UDP: 0.11%; broadcast PU: 0.33%)^37^. Despite the small increase in N_2_O emissions, the contribution of N_2_O (196 and 138 kg CO_2_ eq ha^−1^, respectively) to total GHG emissions (methane [CH_4_] +N_2_O) from rice fields is fairly small (<5%) because of the higher (>95%) contribution of CH_4_, a major GHG emitted from rice fields (but not measured in this study)^58^. AWD irrigation can drastically reduce CH_4_ emissions, which offsets increased N_2_O emissions. Overall, AWD irrigation reduces GHG (CH_4_ and N_2_O) emissions from rice fields by up to 40%^58^. Therefore, efficient N management strategies, such as UDP with AWD irrigation, can contribute to mitigating the negative environmental effects of N fertilizer while permitting savings on fertilizers and increasing crop yields.

## Methods

### Experimental site and climate

The field experiments were conducted at the Bangladesh Rice Research Institute (BRRI), Gazipur, Bangladesh (latitude: 23° 59ʹ 25ʹʹ, longitude: 90° 24ʹ 33ʹʹ), during Boro 2015 and Boro 2016 under an AWD irrigation regime. Boro is the dry season irrigated rice grown between December/January and April/May. The physicochemical properties of the soil before the start and after completion of the experiments are presented in Table 4. The daily average air temperature and rainfall throughout the rice-growing period in Boro 2015 and Boro 2016 are shown in Fig. 2a,b. The minimum air temperatures during both years were similar, ranging from 13.9 °C (2015) to 15.0 °C (2016) in January, while maximum air temperatures recorded in April were 29.3 °C in 2015 and 32.5 °C in 2016. The mean annual air temperatures were 22.7 °C and 24.5 °C in 2015 and 2016, respectively. During the experimental period, maximum rainfall occurred in March and April for both years (Fig. 2a,b).

**Table 4:** Physicochemical properties of soils before the experiment and after harvest of Boro 2016.

Soil properties	Initial soil (2013)	After harvest of Boro 2016
pH-H_2_O	5.8	6.2
Organic carbon (%)	1.27	1.26
Total N (%)	0.14	0.12
Available P (mg kg^−1^)	11.47	11.37
Available K (cmol_c_ kg^−1^)	0.12	0.10
Texture	Clay loam	

### Experimental design and treatments

The three N fertilizer treatments used in the studies were: (i) control, 0 kg N ha^−1^; (ii) UDP, deep placement of urea briquettes at 78 kg N ha^−1^; and (iii) PU, broadcast application of prilled urea at 104 kg N ha^−1^. The N rates and placement methods selected for this study are based on existing N management practices in Bangladesh. UDP increases NUE by 30–35% over broadcast application of PU and increases grain yield up to 20%. Therefore, the N rate for UDP was 25% less than with the broadcast PU (recommended dose)^17–20,59^. Treatments were arranged in a randomized complete block design, with three replications. Each experimental plot was 4.8 m × 3.2 m. Urea briquettes of 2.7 g were deep-placed in a reduced zone (7–10 cm depth) at 40 cm × 40 cm spacing between four hills of rice at each alternate row eight to 10 days after transplanting (DAT). A total of 62,500 briquettes per hectare were used, supplying 78 kg N ha^−1^. Urea briquettes were applied as a single application during the first topdressing of PU, while PU was broadcast in three splits at 10 DAT, maximum tillering, and panicle initiation stages.

### Crop management

Phosphorus (triple superphosphate) and potassium (muriate of potash) fertilizers at 25 kg P ha^−1^ and 85 kg K ha^−1^, respectively, were applied during final land preparation as basal applications to all plots. In addition, sulfur (S) and zinc (Zn) were applied to all plots as basal applications at the rate of 15 kg S ha^−1^ as gypsum and 3 kg Zn ha^−1^ as zinc sulfate. Two to three rice (variety: BRRI dhan 28) seedlings per hill were transplanted with a spacing of 20 cm × 20 cm.

For irrigation, perforated PVC pipe was inserted (15 cm depth) in each plot at 10 DAT. Floodwater depth inside the PVC pipe was monitored daily, and plots were irrigated when water depth in AWD pipes was 12–15 cm below the soil surface (Fig. 2c,d). In 2015, AWD was successfully accomplished (−15 cm from the soil surface) four times, while in 2016 it was not successfully accomplished (−5 to −10 cm) during each dry period due to the poor drainage system. However, in 2016, during two drying periods, a water table level very close to 15 cm below the soil surface was accomplished immediately before harvesting (Fig. 2c,d). During the rest of the drying period, the water table was 5–10 cm below the soil surface. AWD treatment started at 25 DAT and continued until two weeks before harvesting. However, irrigation was provided regularly during fertilizer application and during the flowering stage.

### Measurement of N_2_O and NO emissions

An automated closed chamber technique was used for the measurement of N_2_O and NO gases. A detailed description of this system was reported in our previous study^37^. Briefly, the automated N_2_O and NO gas measurement system consists of 12 plexiglass gas chambers. Each chamber was installed in the respective plot between two rows of rice, leaving four border rows over a fixed aluminum base, which covered a surface area of 0.148 m^2^ and a headspace volume of 0.0578 m^3^ (57.8 liters). In 2015, nine chambers were installed under AWD and three under CSW (non-replicated, not reported herein). In 2016, the three chambers used under CSW irrigation were moved to UDP plots. Each UDP plot had two chambers – one in a fertilized row and another in an unfertilized row.

Six air samples were taken at eight-minute intervals (0, 8, 16, 24, 32, and 40 minutes [min]) in each three-hour sampling sequence. Within a three-hour cycle, a gas chamber was closed for 40 min for gas sampling. One sample was collected just before closing the chamber, which represents the ambient air. Each gas chamber was sampled at three-hour intervals (i.e., eight samples per day). There were three sets of four chambers, one set for one replication (i.e., four chambers were sampled in a one-hour cycle). The three-hour interval consisted of three one-hour cycles: first one-hour cycle – chambers 1–4 (Rep 1); second one-hour cycle – chambers 5–8 (Rep 2); and third one-hour cycle – chambers 9–12 (Rep 3). Chambers 4, 8, and 12 represent the non-replicated treatments of the CSW experiment (for 2015).

Gas samples collected from the chambers were passed to the analyzers using a Teflon tube with a 0.25-in diameter via a 13-port sample manifold (equipped with a solenoid valve, i.e., KIP valve). Of 13 valves in the sample manifold, 12 were used for air samples of the respective 12 chambers, while the last valve was used for the calibration gases (i.e., connected to the calibrator). Each sample valve in the manifold was controlled by a datalogger (Campbell Scientific CR3000) via 16-port (channel) relay controller (SDMCD16 AC/DC Relay Controller). The sample flow rate is determined by the vacuum pump and flow controller of the respective gas analyzers. The sample flow rates for NO and N_2_O measurements were ca. 450 cm^3^ min^−1^ and ca. 750 cm^3^ min^−1^, respectively. Air samples were filtered and dried before passing to the respective analyzers.

N_2_O concentration of the air sample was measured by a Teledyne Advanced Pollution Instrumentation (API) T320U Gas Filter Correlation Analyzer, which uses the infrared absorption principle. NO concentration of the air sample was measured by a Teledyne API T200 Nitrogen Oxide Analyzer. Both N_2_O and NO analyzers were calibrated weekly using a Teledyne T700 Dynamic Dilution Calibrator. The NO and N_2_O analyzers were calibrated for two ranges of concentration (i.e., the low range for the NO and N_2_O analyzers was 40 ppb and 1,600 ppb, respectively, and the high range was 400 ppb and 8,000 ppb, respectively). T320U (N_2_O analyzer) can analyze N_2_O concentration up to 200 ppm, with a lower detection limit of 10 ppb. Similarly, the T200 (NO analyzer) can analyze NO concentration up to 20 ppm, with a lower detection limit of 0.4 ppb.

The concentrations of N_2_O and NO increased or decreased during chamber closure time. Therefore, the N_2_O and NO fluxes were calculated from the slope of the linear regression curve on concentration of respective gases against chamber closure time. An emission event was considered significant when the slope was significant at P < 0.05 and the R^2^ value was 0.65 or higher. Hence, flux rates where R^2^ < 0.65 were not considered emission events and were discarded. The slope (ppb min^−1^) from the significant emission events was corrected for air temperature, atmospheric pressure, and the ratio of chamber volume to surface area using the following formula:

$$\begin{array}{c}{\rm{Emission}}\,{\rm{rate}}\,(\mu g\,{{\rm{N}}}_{{\rm{2}}}{\rm{O}}\mbox{--}{\rm{N}}\,{\rm{or}}\,{\rm{NO}}\mbox{--}{\rm{N}}\,{{\rm{m}}}^{\mbox{--}2}\,{{\rm{h}}}^{\mbox{--}1})\\ \,=\frac{ppb\,{\min }^{-1}\times V\times MW\times SP\times 60}{[0.08206\times {(273+T)}^{0}K]\times A\times 1,013.25\times 1,000}\end{array}$$

Where,

V is the volume of the gas chamber in L (57.8).

MW is the molecular weight of the respective gas in ng nmol^−1^.

(NO–N: 14, N_2_O–N: 28).

SP is the observed atmospheric pressure in millibars (mbar).

60 is the conversion factor for time (min to h).

0.08206 is the gas law constant (L atm mol^−1^ °K^−1^).

T is the temperature inside the chamber (°C).

A is the area covered by chamber (0.148 m^2^).

1,013.25 is the standard pressure in mbar.

1,000 is the conversion factor for mass (ng to µg).

Cumulative seasonal total emissions (g N_2_O-N or NO-N) were calculated summing the hourly emission rates. Yield-scaled N_2_O and NO emissions (g N t^−1^ grain) were calculated from the ratio of seasonal total emissions to grain yields.

### Data analysis

Analysis of variance (ANOVA) of seasonal cumulative emissions, yield-scaled emissions, and EFs of N_2_O and NO gases, grain yield, total N uptake, and NUE was determined with SAS 9.3, Generalized Linear Mixed Models. Treatment, year, and their interaction (T × Y) were handled as fixed effects and error term – Rep (T × Y) – was considered a random effect. A pairwise comparison of treatment means was conducted with Tukey’s honest significant difference (HSD) test at a 5% level of probability.

## Conclusions

UDP in rice fields under AWD irrigation increased NUE and grain yields significantly (p < 0.05) compared to broadcast PU. UDP had discernible, but variable, effects on N_2_O emissions. N_2_O emission peaks were observed with both UDP and broadcast PU in each dry period. But under flooding conditions, N_2_O emission peaks were found only after broadcast application of PU. However, cumulative seasonal N_2_O emissions with UDP and broadcast PU were similar. Similarly, NO emissions were very minimal, compared to N_2_O emissions, and inconsistent for both UDP and PU treatments. Our results confirm that UDP with AWD irrigation is equally effective as UDP with CSW irrigation, since it increased grain yields and NUE compared to broadcast PU without significant (p > 0.05) effects on N_2_O and NO emissions.
